# Pregnancy-Induced Changes in Systemic Gene Expression among Healthy Women and Women with Rheumatoid Arthritis

**DOI:** 10.1371/journal.pone.0145204

**Published:** 2015-12-18

**Authors:** Anuradha Mittal, Lior Pachter, J. Lee Nelson, Hanne Kjærgaard, Mette Kiel Smed, Virginia L. Gildengorin, Vibeke Zoffmann, Merete Lund Hetland, Nicholas P. Jewell, Jørn Olsen, Damini Jawaheer

**Affiliations:** 1 Children's Hospital Oakland Research Institute, Oakland, California, United States of America; 2 Department of Mathematics, University of California, Berkeley, California, United States of America; 3 Fred Hutchinson Cancer Research Center, Seattle, Washington, United States of America; 4 University of Washington, Seattle, Washington, United States of America; 5 Juliane Marie Center, Copenhagen University Hospital, Rigshospitalet, Copenhagen, Denmark; 6 DANBIO Registry and Copenhagen Centre for Arthritis Research, Centre for Rheumatology and Spine Diseases VRR, Rigshospitalet, Copenhagen, Denmark; 7 Department of Clinical Medicine, Faculty of Health and Medical Sciences, University of Copenhagen, Copenhagen, Denmark; 8 School of Public Health, University of California, Berkeley, California, United States of America; 9 School of Public Health, University of California Los Angeles, Los Angeles, California, United States of America; 10 Department of Clinical Epidemiology, Aarhus University Hospital, Aarhus, Denmark; Queen's University Belfast, UNITED KINGDOM

## Abstract

**Background:**

Pregnancy induces drastic biological changes systemically, and has a beneficial effect on some autoimmune conditions such as rheumatoid arthritis (RA). However, specific systemic changes that occur as a result of pregnancy have not been thoroughly examined in healthy women or women with RA. The goal of this study was to identify genes with expression patterns associated with pregnancy, compared to pre-pregnancy as baseline and determine whether those associations are modified by presence of RA.

**Results:**

In our RNA sequencing (RNA-seq) dataset from 5 healthy women and 20 women with RA, normalized expression levels of 4,710 genes were significantly associated with pregnancy status (pre-pregnancy, first, second and third trimesters) over time, irrespective of presence of RA (False Discovery Rate (FDR)-adjusted p value<0.05). These genes were enriched in pathways spanning multiple systems, as would be expected during pregnancy. A subset of these genes (n = 256) showed greater than two-fold change in expression during pregnancy compared to baseline levels, with distinct temporal trends through pregnancy. Another 98 genes involved in various biological processes including immune regulation exhibited expression patterns that were differentially associated with pregnancy in the presence or absence of RA.

**Conclusions:**

Our findings support the hypothesis that the maternal immune system plays an active role during pregnancy, and also provide insight into other systemic changes that occur in the maternal transcriptome during pregnancy compared to the pre-pregnancy state. Only a small proportion of genes modulated by pregnancy were influenced by presence of RA in our data.

## Background

Pregnancy is known to have beneficial effects on rheumatoid arthritis (RA) in a number of ways. New onset of RA is rare during the gestational period, suggesting that pregnancy may be protective against development of RA [1–3]. Pregnancy even appears to protect against RA onset beyond the gestational period in the form of vaccine-like protection [4]. Further, women with RA often experience a natural and dramatic improvement in disease activity during pregnancy [5–8]. Some pregnancy-related factors that have been associated with the pregnancy-induced improvement of RA disease activity include maternal-fetal HLA disparity [9] and microchimerism [10]. The mechanism(s) behind the protective effects of pregnancy are, however, not entirely clear. While there is no doubt that pregnancy induces drastic biological changes systemically, the specific systemic changes that occur as a result of pregnancy and their effects on maternal health have not been thoroughly examined. Hence, as a first step in examining the influence of pregnancy on RA, it is important that we gain a better understanding of systemic biological changes associated with healthy pregnancy and that we determine whether pregnancy-induced biological changes are altered by the presence of RA, irrespective of disease activity levels.

Global gene expression studies are well-suited to inform us on such changes that occur during pregnancy. However, surprisingly little is known about pregnancy-induced systemic changes in gene expression among healthy women because most gene expression studies in pregnancy have focused on the maternal-fetal interface to understand how fetal tolerance is established and maintained [11–13]. There have been very few gene expression studies on RA pregnancy [14–17], all of which were focused on pregnancy-induced changes in disease activity. Thus, it still remains to be determined whether systemic biological changes associated with pregnancy can be altered by the presence of RA, irrespective of disease activity levels.

In the present study, we have examined the hypothesis that the maternal immune system is active during pregnancy among both healthy women and women with RA. We have examined global gene expression profiles among 5 healthy women and 20 women with RA from pre-pregnancy to the third trimester, to identify genes that exhibit pregnancy-induced changes in expression. We also examined whether the presence of RA (irrespective of level of disease activity) influences pregnancy-associated gene expression.

## Results

### Study subjects

Overall, age at conception was similar among healthy women and women with RA: healthy (mean±SD): 31.4 ± 5.8 years; RA: 31.6 ± 4.6 years (p = 0.99). The women with RA had the disease for a mean duration of 5.1 ± 3.3 years.

Medication use: Among the women with RA who had complete follow up (n = 13), 2 did not take any medications while in the study, 7 took prednisolone and/or sulfasalazine before and/or during pregnancy, and 4 took anti-TNF agents before pregnancy and in the first trimester. Of the women on prednisolone and/or sulfasalazine, one took methotrexate before pregnancy, and one was on hydroxychloroquine throughout pregnancy. The 7 women with incomplete follow up took prednisolone or sulfasalazine (n = 6) or were not on medication (n = 1).

### Data quality

The average number of paired-end reads across all samples was 73 million. Of these, an average of 40% mapped to the reference transcriptome (S1 Fig). No significant differences were observed in number of mapped reads across time points (ANOVA, p = 0.93). Sample replicates processed in separate batches achieved a Pearson correlation of at least 0.97, following correction for batch effects (S2 Fig). After filtering out genes with low expression across all samples and genes differentially expressed between replicate samples across batches, a total of 13,655 genes (39%) remained for downstream analyses.

### Genes modulated by pregnancy in healthy women and women with RA

**Fig 1:**
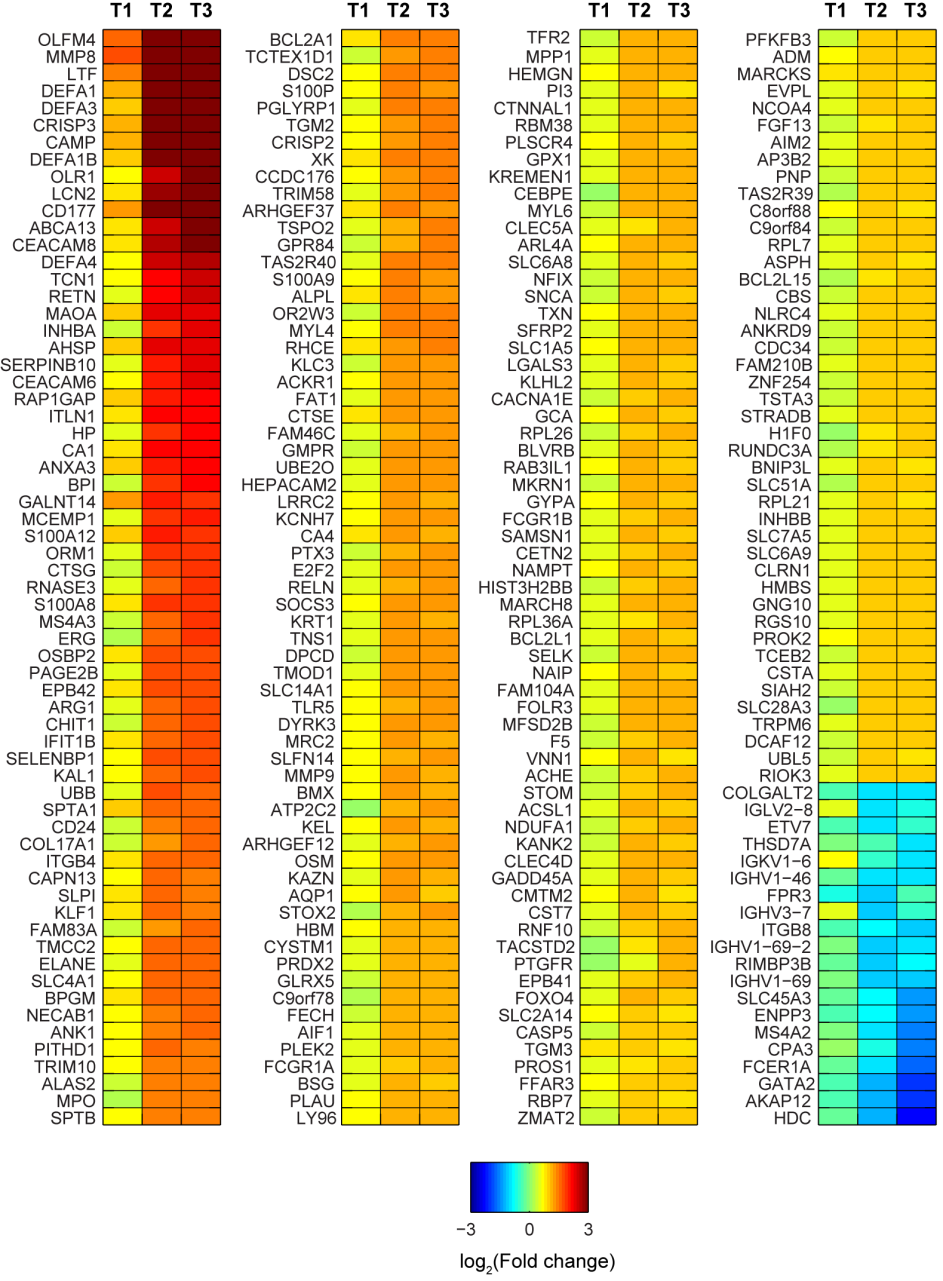
Heatmap showing temporal trends in expression among genes with pregnancy-associated expression, compared to pre-pregnancy levels in healthy women. Log-transformed (log_2_) values of the average fold change in expression compared to pre-pregnancy levels are plotted. Only genes that exhibited 2-fold or higher change in expression (compared to healthy pre-pregnancy baseline levels) in any trimester are shown. These patterns were similar in healthy women and in women with RA.

In the GEE models, of 13,655 genes analyzed, 4,710 had expression levels associated with pregnancy status over time (q<0.05), irrespective of whether a woman did or did not have RA. Pathway analysis indicated that many of these genes were enriched pathways for: genetic information processing, metabolism, signal transduction, cellular processes and organismal systems (immune, endocrine, excretory, nervous), development, and disease pathways (Table 1). Among the genes with pregnancy-associated expression, 256 genes showed greater than two-fold change in expression levels during pregnancy compared to pre-pregnancy levels among healthy women. These had distinct temporal trends, with most being up-regulated as pregnancy progressed and others showing reduced expression with advancing pregnancy stages (Fig 1). Significant changes in the expression profiles compared to pre-pregnancy were observed during second trimester and were maintained during third trimester. Examples of genes involved in immune system processes and defense response that displayed a marked pregnancy-related up-regulation included: OLFM4, MMP8, LTF, DEFA1, DEFA3, DEFA1B, CRISP3, CAMP, OLR1, LCN2, CD177, ABCA13 and CEACAM8. Furthermore, genes involved in mast cell activation and immunoglobulin binding were significantly downregulated in the second and third trimesters. Two such significant transcripts were the Fc fragment of immunoglobulin Epsilon Receptor (FCER1A) and Membrane Spanning 4-domains subfamily A member 2 (MS4A2).

**Table 1:** Significantly enriched KEGG pathways among 4,710 genes associated with pregnancy.

	KEGG pathways			Genes identified	q value*
				(Genes in pathway)	
**Pathways enriched in genes up-regulated during pregnancy**					
	**Genetic information processing**				
		*Translation*			
			Ribosome	51 (89)	7x10^-24^
		*Folding*, *sorting and degradation*			
			Ubiquitin mediated proteolysis	30 (135)	2x10^-3^
			Protein processing in endoplasmic reticulum	30 (165)	0.02
			Proteasome	11 (44)	0.03
		*Replication and repair*			
			Non-homologous end joining	5 (13)	0.04
		*Transcription*			
			Spliceosome	24 (127)	0.03
	**Metabolism**				
		*Metabolism*			
			Metabolic pathways	169 (1130)	5x10^-4^
		*Energy metabolism*			
			Oxidative phosphorylation	31 (132)	6x10^-4^
		*Metabolism of other amino acids*			
			Glutathione metabolism	13 (50)	0.02
		*Carbohydrate metabolism*			
			Amino sugar and nucleotide sugar metabolism	14 (48)	4x10^-3^
			Inositol phosphate metabolism	14 (57)	0.02
			Galactose metabolism	8 (27)	0.03
			Pentose phosphate pathway	8 (27)	0.03
	**Signal transduction**				
		*Signal transduction*			
			MAPK signaling pathway	53 (268)	4x10^-4^
			mTOR signaling pathway	14 (52)	8x10^-3^
			Jak-STAT signaling pathway	30 (155)	0.01
			Phosphatidylinositol signaling pathway	16 (78)	0.04
	**Cellular processes**				
		*Transport and catabolism*			
			Phagosome	39 (152)	2x10^-5^
			Lysosome	27 (121)	3x10^-3^
			Endocytosis	46 (201)	4x10^-5^
			Regulation of autophagy	9 (34)	0.04
		*Cell motility*			
			Regulation of actin cytoskeleton	41 (212)	3x10^-3^
		*Cell growth and death*			
			Apoptosis	20 (87)	8x10^-3^
	**Organismal processes**				
		*Immune system*			
			Fc gamma R-mediated phagocytosis	29 (94)	1x10^-5^
			Toll-like receptor signaling pathway	30 (102)	2x10^-5^
			NOD-like receptor signaling pathway	17 (58)	1x10^-3^
			B cell receptor signaling pathway	19 (75)	3x10^-3^
			Leukocyte transendothelial migration	24 (116)	0.01
			Fc epsilon RI signaling pathway	18 (79)	0.01
			Hematopoietic cell lineage	19 (88)	0.02
			T cell receptor signaling pathway	21 (108)	0.03
			Chemokine signaling pathway	32 (183)	0.04
			Natural killer cell mediated cytotoxicity	24 (136)	0.05
		*Development*			
			Osteoclast differentiation	44 (128)	2x10^-10^
		*Endocrine system*			
			Insulin signaling pathway	33 (138)	3x10^-4^
			Adipocytokine signaling pathway	18 (68)	3x10^-3^
		*Excretory system*			
			Collecting duct acid secretion	10 (27)	3x10^-3^
		*Nervous system*			
			Neurotrophin signaling pathway	28 (127)	3x10^-3^
			Long-term potentiation	15 (70)	0.03
	**Disease pathways**				
		*Infectious diseases*: *Bacterial*			
			Epithelial cell signaling in Helicobacter pylori infection	19 (68)	1x10^-3^
			Vibrio cholerae infection	13 (54)	0.02
			Shigellosis	14 (61)	0.03
			Bacterial invasion of epithelial cells	15 (70)	0.03
		*Infectious diseases*: *Parasitic*			
			Leishmaniasis	21 (72)	4x10^-4^
			Chagas disease	25 (104)	1x10^-3^
			Toxoplasmosis	29 (132)	2x10^-3^
		*Cancers*			
			Renal cell carcinoma	20 (70)	7x10^-4^
			Pathways in cancer	55 (326)	7x10^-3^
			Endometrial cancer	13 (52)	0.02
			Acute myeloid leukemia	14 (57)	0.02
			Non-small cell lung cancer	13 (54)	0.02
			Glioma	15 (65)	0.02
			Chronic myeloid leukemia	16 (73)	0.03
			Pancreatic cancer	15 (70)	0.03
			Pancreatic cancer	15 (70)	0.03
			Thyroid cancer	8 (29)	0.04
		*Neurodegenerative diseases*			
			Alzheimer's disease	40 (167)	5x10^-5^
			Huntington's disease	41 (183)	2x10^-4^
			Amyotrophic lateral sclerosis	15 (53)	4x10^-3^
			Parkinson’s disease	27 (130)	7x10^-3^
		Immune diseases			
			Rheumatoid arthritis	18 (91)	0.04
		Endocrine and metabolic diseases			
			Type II diabetes mellitus	11 (48)	0.05
**Pathways enriched in genes downregulated during pregnancy**					
	**Metabolism**				
		*Glycam biosynthesis and metabolism*			
			N-Glycan biosynthesis	17 (49)	2x10^-3^
	**Disease pathways**				
		*Immune diseases*			
			Primary imunodeficiency	19 (35)	2x10^-7^

* q value refers to the FDR-adjusted p value associated with the KEGG pathways enriched in pregnancy-associated genes. Pathways are grouped into broad functional categories.

### Genes with pregnancy-associated expression modified by presence of RA

**Fig 2:**
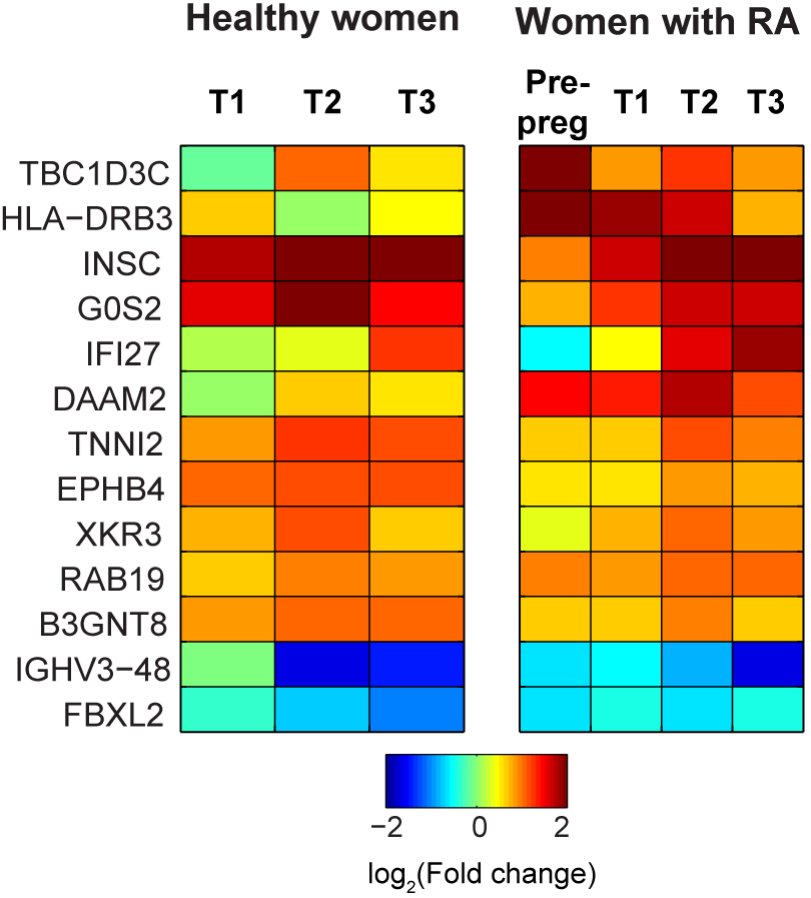
Heatmap showing genes with pregnancy-associated expression patterns that are altered by presence of RA. Log-transformed (log_2_) values of the average fold change in expression compared to baseline (i.e. pre-pregnancy levels among healthy women) are plotted for healthy women and for women with RA. Only genes that exhibited 2-fold or higher change in expression (compared to healthy pre-pregnancy baseline levels) in any trimester are shown.

In contrast to the genes with pregnancy-associated expression irrespective of the presence or absence of RA, another 98 genes exhibited expression patterns during pregnancy that were dependent on both pregnancy status and presence or absence of RA; these were identified through the interaction term (RA×Pregnancy) in the GEE models. Functional classification using the GO database revealed that these genes were involved in a variety of biological processes including regulation of immune system (Table 2). Of these, thirteen genes displayed greater than two-fold change in expression before and/or during pregnancy, compared to pre-pregnancy levels among healthy women (Fig 2).

**Table 2:** Functional categories of genes with expression patterns differentially associated with pregnancy in presence or absence of RA.

Biological process / Molecular function	Genes
Regulation of immune system process	HLA-DRB3, *IL15*
Metabolic process	*ACAD11*, DECR2, LCAT, *PKD2*, PNPLA2, *IL15*, CAMKK1
Regulation of catalytic activity	*PKD2*, IFI27, CAMKK1
Hormone transport	HLA-DRB3, *CACNA1C*
Cellular response to osmotic stress	*PKD2*, RCSD1
Neurogenesis	MAP1S, *CTHRC1*, *CACNA1C*, *USH2A*, ARHGDIA
Tissue morphogenesis	*PKD2*, *CTHRC1*, CCM2
Voltage-gated calcium channel activity	*PKD2*, *CACNA1C*
Endothelial cell migration	EPHB4, HDAC5
Protein binding	TNNI2, *CACNA1C*, RPH3AL, *USH2A*, DAAM2, RCSD1, MAP1S, PKD2, FAM101B, MYO7B, SH2D4A, SYTL2
Cofactor binding	*SQLE*, *ACAD11*, HSD11B2, *MTO1*
Hydrolase activity	CES2, HDAC5, LCAT, PNPLA2

Genes up-regulated in healthy pregnancy are shown in normal font, genes downregulated in healthy pregnancy are shown in italics, genes up-regulated in RA pregnancy are not underlined and genes downregulated in RA pregnancy are underlined.

### Genes with expression levels associated with presence of RA and/or medication use

One hundred and eleven genes had expression levels that were associated with presence of RA in the GEE models (q<0.05), irrespective of pregnancy status. The pathways and/or biological processes that these genes are involved in are shown in Table 3. Our analyses also detected genes with expression levels influenced by medication use; these were involved in immune response, defense response, RNA binding, and transferase activity.

**Table 3:** KEGG pathway and Gene Ontology Biological process categories of the genes showing association with RA.

Pathway / biological process	Genes
mTOR signaling pathway	ULK1, *AKT1*
Toll-like receptor signaling pathway	IKBKG, *AKT1*, TOLLIP
Metabolic pathways	MAN2A2, GAA, *GALC*, CSGALNACT1, MAOA, NDUFV3, ABAT, *LIPC*, *DBT*, PISD
Fatty acid metabolic process	*FADS1*, *FADS2*, FADS3, ABAT, *AKT1*, *LIPC*, *SCAP*
Cellular polysaccharide metabolic process	GAA, *AKT1*, PYGM, CSGALNACT1
Intracellular transport of viral material	VPS37C, VPS37B
Neurotransmitter catabolic process	MAOA, ABAT
t-RNA binding	*SLFN11*, IGHMBP2
DNA helicase activity	IGHMBP2, *HELB*
Catalytic activity	GAA, TUBB2A, PYGM, SPTSSB, ACAP1, ULK1, ERCC4, *RNF43*, IGHMBP2, ABAT, GRK6, *KIF21B*, PISD, *SPPL2A*, FADS3, ECHDC3, TPST1, NDUFV3, DBT, UBE2M, CARS2, *DCTN1*, PLK3, FADS1, FADS2, *AKT1*, ADCK3, *GALC*, *HELB*, UFSP1, PPM1F, USP10, PGLYRP1, *LIPC*, *ALK*, MAN2A2, PRDX5, MAOA, CSGALNACT1, DTD1, *TRIM36*
GTPase activator activity	ARHGAP9, GMIP, ARHGAP27, ACAP1, RAP1GAP2

Up-regulated genes are shown in normal font and downregulated genes are in italics.

## Discussion

Using samples from an ethnically homogeneous pregnancy cohort followed in real time from pre-pregnancy, we have examined changes in global gene expression among healthy women and women with RA using RNA-seq technology. Given that pregnancy-induced gene expression could not previously be examined relative to the pre-pregnancy state, our findings are novel. We have identified genes that demonstrate altered expression during pregnancy compared to the pre-pregnancy baseline, and shown how their temporal patterns of expression change throughout pregnancy. These include several potentially novel genes that are at least two-fold differentially expressed during pregnancy compared to before pregnancy. In addition, we have also identified genes that demonstrate different temporal patterns of expression through pregnancy between healthy women and women with RA; that is, their pregnancy-associated expression patterns were altered by whether a woman was healthy or had RA.

Human pregnancy is known to induce extensive physiological changes in the mother, involving almost every system. Our results at the gene expression level fit well in this context, indicating systemic changes in maternal transcriptome spanning multiple cellular and organismal systems, pathways and biological processes during pregnancy. Among the pathways that we identified as being enriched in genes with pregnancy-associated expression, several were related to immune function. This is especially relevant since pregnancy is known to be associated with immunological changes and challenges, not just locally at the maternal-fetal interface, but also at the systemic level. The pathways identified relate to natural killer cell mediated cytotoxicity, and signaling pathways involving Toll-like receptor, NOD-like receptor, T cell receptor, and B cell receptor, suggesting that both innate and adaptive immune responses play a role in pregnancy. Further, several of the genes that were the most highly up-regulated during pregnancy are expressed in neutrophils, in line with previous reports of neutrophil activation during pregnancy [18–20]. An overexpression of neutrophil-related genes, namely OLFM4, MMP8, DEFA1 and CEACAM8, during pregnancy has also been reported by Heung *et*. *al*. [21]. Our findings thus add support to mounting evidence that the maternal immune system is active, and not generally immuno-suppressed during pregnancy [22].

Previous studies of systemic gene expression changes that occur in the mother during healthy pregnancy have been few and the findings inconsistent. Some studies reported no significant differences between pregnancy and post-partum expression profiles of healthy women [14, 17]. Other reports share some degree of overlap in genes/pathways that we have identified as being modulated by pregnancy. For example, a study of peripheral blood mononuclear cells (PBMC) profiles using microarray data identified genes differentially expressed during and after pregnancy [16]. Pathways that overlapped with our findings included those for: apoptosis, cancer, Fc gamma R-mediated phagocytosis, natural killer cell-mediated cytotoxity, and signaling pathways involving MAPK, T cell receptor, Toll-like receptor, and adipocytokine. In a longitudinal study of 11 women followed from the first trimester through 6 weeks post-partum, pairwise analyses of microarray and RNA-seq data from cell-free plasma RNA identified 16 genes in common with our findings [23]. Of interest, several genes such as MMP8, S100P, LIN7A, ZNF438, TCN1, B3GNT5, PLEK2 and PAPPA showed similar temporal changes in expression as pregnancy progressed as observed in our study. A small (n = 4) study of cell-free plasma comparing RNA-seq data from before and after delivery identified several genes with pregnancy-associated expression that were also found in our data [24].

The inconsistencies in findings between these previous studies and our study may be accounted for by a number of factors. First, whilst these studies used either cell-free sera or PBMC as the source of total RNA, our source of total RNA included both the cellular and cell-free fractions of whole blood. Second, these few studies have identified pregnancy-related changes using postpartum data as baseline. However, the postpartum state is itself associated with major changes such as lactation, increased risk of some cancers [25], depression [26, 27], and significant immunologic changes as reflected by increased risk of some autoimmune diseases [1, 28, 29]. Therefore, the appropriate baseline when identifying pregnancy-specific changes is the pre-pregnancy state, which was not available in these studies. Third, we used GEE models to make the most of the data available at all time points. Pairwise analyses of data available from the same subjects across time points [23, 24] limit the power of the analyses. Additionally, in cases where data on each woman was available at only one time-point (i.e. cross-sectional data) [16], changes identified between time points may have been in part due to between-subject variability. Fourth, while several of the previous studies were based on microarray data [17, 23], we used the more accurate and reliable RNA-seq technology [30, 31].

Our findings relating to genes that have pregnancy-related patterns altered by presence of RA are novel. Several of the genes identified have previously been implicated in RA. For example, HLA-DRB3 and G0/G1 Switch 2 (G0S2) expression have been reported to predict response to anti-TNF therapy [32, 33], while Interferon, Alpha-Inducible Protein 27 (IFI27) and Troponin I type 2 (TNNI2) appear to be involved in RA onset or progression [34, 35]. To our knowledge, it had thus far not been demonstrated that these genes can be modulated by both RA and pregnancy status. It is not clear yet whether and how the remaining genes that exhibited pregnancy-related patterns altered by presence of RA, may be involved in the disease.

Our study does have strengths as well as limitations. The sample size was small, but longitudinal samples available from the majority of subjects enabled us to use GEE models to enhance power and to eliminate noise due to between-subject variability and time-stable confounders. The ethnic homogeneity of our study population also was an advantage. We cannot eliminate the possibility of technical bias and/or batch effects having been introduced in the data. However, we randomized sample order prior to any sample processing, used a block design for sequencing, and at the data processing step, we used sample replicates to assess and mitigate batch effects. We also adjusted for any residual batch effects in the statistical models. Although we adjusted for medication use in the model, we did not adjust for specific medications that may have an effect on the immune system and/or dosage due to the heterogeneity in medication use. Because our goal was to identify overall systemic gene expression changes resulting either from altered expression of specific genes or from changes in cell proportions, we also did not examine whether proportions of different cell types in blood samples changed across time points. We nevertheless adjusted for medication use in the analysis, which should have corrected for drug-induced changes in cell type proportions. The use of total RNA from whole blood may also mean that expression profiles of neutrophils may have dominated a large part of the observed expression patterns. Some genes expressed by neutrophils such as MMP8, LTF, CRISP3, CD177 and DEFA4 did exhibit significant changes in expression during the course of pregnancy since neutrophils appear to have an active role in later stages of pregnancy and in labor [36]. However, the sensitivity of RNA-seq technology enabled us to also detect transcripts that were not neutrophil-specific, including those specific to cell types present in low proportions in blood. We have not attempted to separate RA-associated gene expression from disease activity-associated gene expression because the women with RA showed a broad range of disease activity, ranging from remission to high disease activity, both before and during pregnancy. Since the pregnancy-related gene expression changes were obtained by averaging over all RA patients, we do not expect them to have been induced by specific disease activity, but by presence of the disease in general.

## Conclusions

In summary, our findings support the hypothesis that the maternal immune system is active during pregnancy, as depicted by the significant changes in expression of immune response genes in the global transcriptome of both healthy women and women with RA. We have identified several genes that demonstrate pregnancy-associated expression patterns that were similar in healthy women and in women with RA, with only few genes showing altered expression in the presence of RA. These findings broaden our understanding of pregnancy-associated systemic changes in the maternal transcriptome among healthy women and women with RA. While it is not yet known how these changes in gene expression might influence the decreased risk of RA onset during pregnancy or contribute to pregnancy-induced amelioration of RA, these results, though still exploratory, may represent a first step towards elucidating the beneficial influence of pregnancy on RA.

## Methods

### Study subjects

Twenty five women of Danish descent, 5 healthy and 20 with RA, were recruited and enrolled in a pregnancy cohort in Denmark. All 5 healthy women and 14 of the women with RA were enrolled before pregnancy; another 6 women with RA were enrolled after they conceived, in the first (n = 2), second (n = 1) and third (n = 3) trimesters. One of the 14 women with RA enrolled before pregnancy was not followed after she conceived because she had a miscarriage. Study subjects were followed prospectively from the time of enrollment through the third trimester. Subjects with RA fulfilled the 1987 revised American College of Rheumatology criteria for RA [37]. The study was approved by the Ethics Committee for Region Hovedstaden (Denmark), the Danish Data Protection Agency, and the Children’s Hospital Oakland Research Institute Institutional Review Board. All subjects provided written informed consent prior to enrolment.

### Sample collection and processing

Blood samples were drawn into PAXgene RNA tubes at 4 time points: before conception and once every trimester during pregnancy (gestational weeks 6–8, 24 and 32). Data on medication use during the 3 months prior to the blood draw were also collected at the same time points from the subjects with RA. Total RNA was extracted from frozen blood samples using the PAXgene Blood RNA Isolation kit according to the manufacturer’s protocol. RNA integrity was assayed using 2100 Bioanalyzer and D1000 ScreenTape (Agilent Technologies). The Illumina TruSeq RNA sample preparation kit was used to generate barcoded cDNA libraries depleted of ribosomal RNAs (rRNAs) and globin mRNAs. Pooled libraries were sequenced on an Illumina HiSeq2500 instrument to generate on average 60 million paired-end reads of 100 bp length. cDNA library preparation and sequencing were performed in two batches. Sample replicates were included in both batches to assess and correct for batch effects. To minimize confounding from experimental effects introduced by variations in library preparation, flow cells or lanes, sample order was randomized prior to library preparation and a blocking design [38] was adopted for sequencing.

### Bioinformatic analyses

The raw sequence reads (FASTQ format) were aligned to cDNA sequences of the human GRCh38 reference assembly available in Ensembl using the Bowtie2 (v2.2.5) algorithm [39]. The reads were mapped as concordant paired reads with stringent read and reference gap penalties (—rdg 6,5—rfg 6,5—score-min L,-.6,-.4) to report all alignments. The resulting alignments in SAM format were converted to BAM format using SAMtools [40]. Transcript abundances were calculated from the BAM files using eXpress (v1.5.1) with default settings [41]. BioMart [42] annotations were used to map the transcript Ensembl identifiers to their associated gene loci using in-house python scripts. Gene-level quantifications were determined by combining the transcript-level quantifications onto their associated gene loci. To remove unwanted variation in the transcriptome profiles associated with batch effects, genes which showed at least 10-fold difference in expression (FDR-adjusted p-value < 0.05) across two batches in replicate samples were filtered out, and correlations in expression levels between batches were computed. Genes with very low read counts across all samples were also removed. To adjust for variable sequencing depths between samples, the raw gene counts were normalized using a weighted trimmed mean of the log expression ratios (Trimmed Mean of M values [TMM] algorithm) as implemented in the edgeR Bioconductor package [43, 44].

### Statistical analyses

#### Longitudinal regression models

To examine associations between repeated measures of gene expression levels over 4 time points corresponding to different pregnancy status (pre-pregnancy, first, second and third trimesters), Generalized Estimating Equations (GEE) models were fitted, with robust estimation, using normalized gene counts as the outcome variable and pregnancy status as the main explanatory temporal variable. Covariates included in the models were presence of RA (present/absent), medication use before/during pregnancy (yes/no), and sequencing batch (1/2). To determine whether associations between gene expression levels and pregnancy status were modified by the presence of RA, an interaction term (RA×Pregnancy) was also included in the model. These models allowed within-person correlations in the repeated measures of the data to be adjusted for. A negative binomial link function was used to handle the over-dispersion in RNA-seq gene counts. Both independent and exchangeable correlation structures were tested. Correction for multiple testing was achieved using the Benjamini-Hochberg False Discovery Rate (FDR) method [45]. We refer to the FDR-adjusted p values as q values [46] henceforth. A threshold of q<0.05 was used to assess statistical significance of associations tested. All statistical computations were performed using STATA version 13.1 software.

#### Temporal patterns in expression levels

For genes demonstrating statistical association with pregnancy status, temporal variations, i.e. from pre-pregnancy to the third trimester, in gene expression levels (log-transformed normalized gene counts) among healthy women and women with RA were examined using pre-pregnancy expression levels in healthy women as reference. The ratio of average expression levels at each time point relative to the average expression levels in the reference group was evaluated as the mean fold change, and these were plotted as a heat map.

#### Gene ontology and pathway analyses

Sets of genes demonstrating evidence of association with pregnancy status were analyzed for over-representation of biological processes and pathways terms in the Gene Ontology (GO) and KEGG databases using a hypergeometric test implemented in WebGestalt [47]. A q value<0.05 was used to define significant enrichment.

## Supporting Information

S1 Fig Mapped Read Counts. Bar plot showing total number of mapped reads for each sample from healthy women and women with RA.
(TIF)

S2 Fig Scatter plots showing high correlation between replicates of the same biological sample. The plots and correlations shown are representative of 3 independent sets of sample replicates included in the 2 batches of samples, to correct for batch effects. In the left panel, log-transformed* gene-level counts from technical replicates of the same biological sample (sample 1) are plotted against each other. These technical replicates represent independent cDNA libraries prepared within a single batch of samples (batch 1). The Pearson correlation between the within-batch replicates was 0.99. In the right panel, the log-transformed gene-level counts are plotted for sample 1/batch 1 on the x-axis and for a technical replicate of sample 1 prepared as part of a separate batch of samples (replicate 2/batch 2) on the y-axis, after correction for batch effects. The Pearson correlation for these between-batch replicates was 0.98. (* To accommodate genes which had a read count of zero, log(counts+1) was used.)
(TIF)

## Abbreviations

ANOVA: analysis of variance
bp: base pair
cDNA: complementary DNA
FDR: False Discovery Rate
GEE: Generalized Estimating Equations
GO: gene ontology
KEGG: Kyoto Encyclopedia of Genes and Genomes
mRNA: messenger RNA
RA: rheumatoid arthritis
RNA-seq: RNA sequencing
rRNA: ribosomal RNA
TMM: Trimmed Mean of M-values
