# Supplementary figures and images for: Pregnancy-Induced Changes in Systemic Gene Expression among Healthy Women and Women with Rheumatoid Arthritis
Source: PLoS One. 2015 Dec 18;10(12):e0145204. doi: 10.1371/journal.pone.0145204 (PMC4684291; doi:10.1371/journal.pone.0145204)

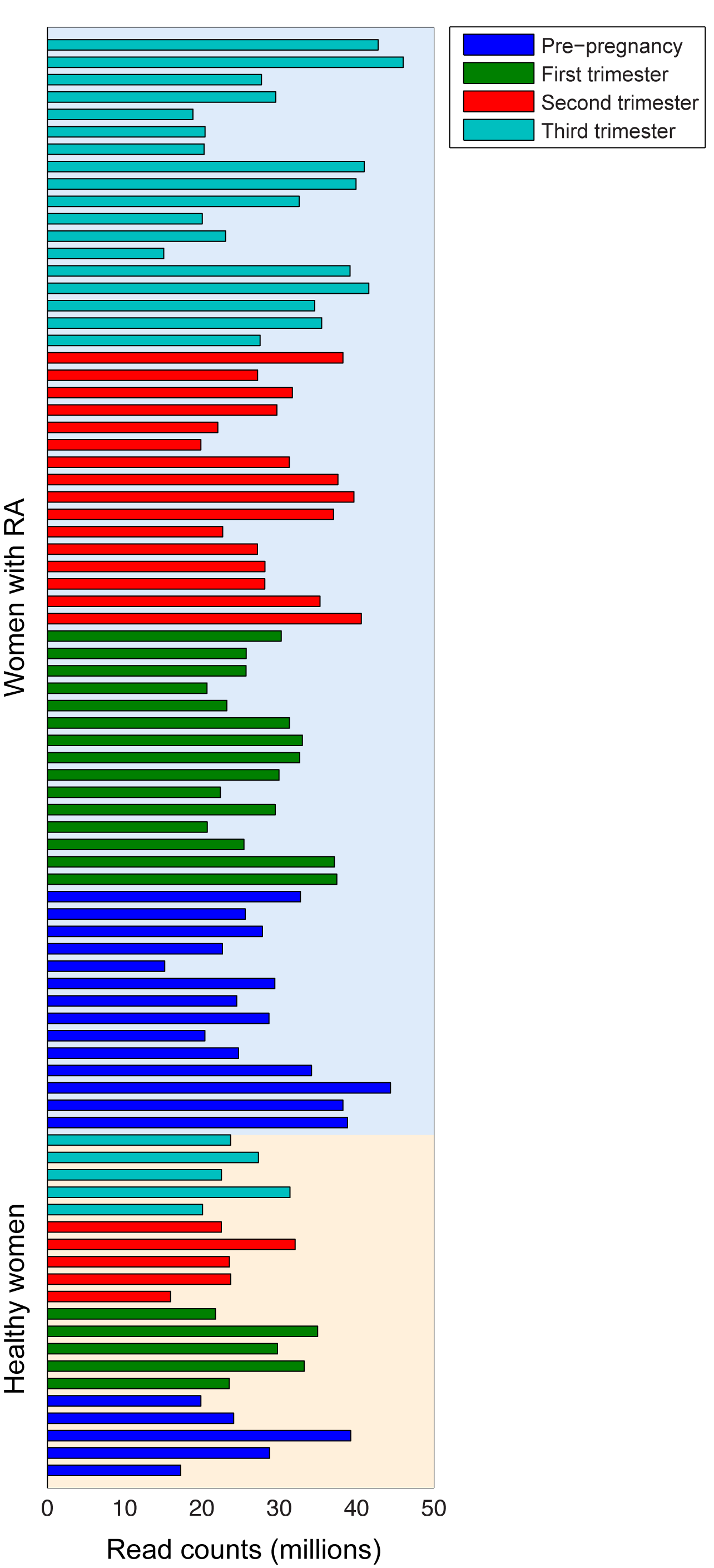

Supplement: S1 Fig — Bar plot showing total number of mapped reads for each sample from healthy women and women with RA. (TIF) [file pone.0145204.s001.tif]

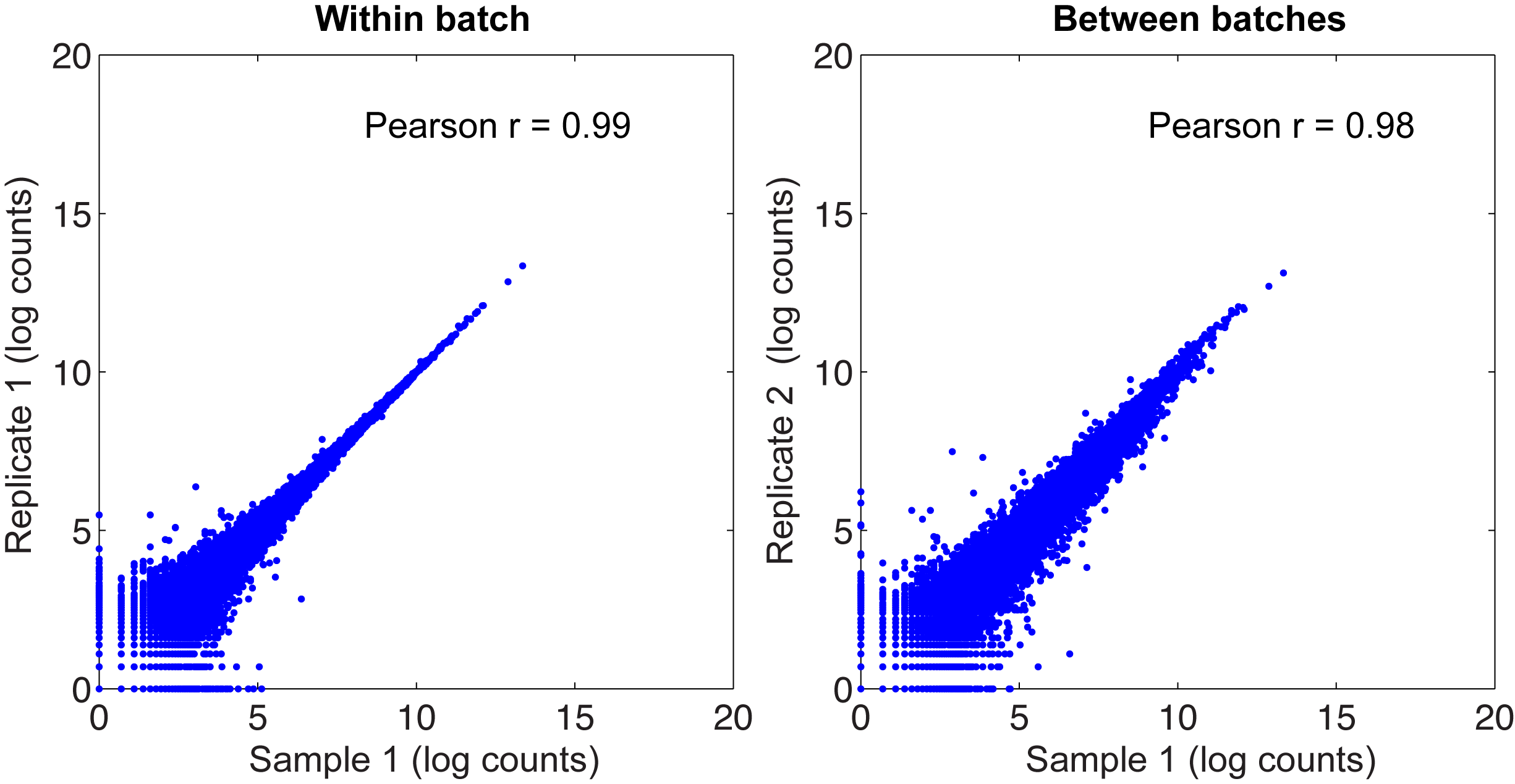

Supplement: S2 Fig — The plots and correlations shown are representative of 3 independent sets of sample replicates included in the 2 batches of samples, to correct for batch effects. In the left panel, log-transformed* gene-level counts from technical replicates of the same biological sample (sample 1) are plotted against each other. These technical replicates represent independent cDNA libraries prepared within a single batch of samples (batch 1). The Pearson correlation between the within-batch replicates was 0.99. In the right panel, the log-transformed gene-level counts are plotted for sample 1/batch 1 on the x-axis and for a technical replicate of sample 1 prepared as part of a separate batch of samples (replicate 2/batch 2) on the y-axis, after correction for batch effects. The Pearson correlation for these between-batch replicates was 0.98. (* To accommodate genes which had a read count of zero, log(counts+1) was used.) (TIF) [file pone.0145204.s002.tif]
